# Menopausal Symptoms among Postmenopausal Women Visiting Outpatient Department of a Tertiary Care Centre: A Descriptive Cross-sectional Study

**DOI:** 10.31729/jnma.7570

**Published:** 2022-07-31

**Authors:** Anjali Subedi, Junu Shrestha, Jainath Kumar Chaudhary

**Affiliations:** 1 Department of Obstetrics and Gynaecology, Manipal College of Medical Sciences, Fulbari, Pokhara, Nepal

**Keywords:** *cross-sectional study*, *menopause*, *prevalence*, *somatic symptoms*

## Abstract

**Introduction:**

Menopause is defined as the complete cessation of menstruation for consecutive 12 months which occurs due to the loss of follicular activity of ovaries from the late 40s to early 50s. The menopausal symptoms are often distressing but lack of awareness leads to failure in addressing the problem. This study aimed to find out the prevalence of menopausal symptoms among postmenopausal women visiting the outpatient department of a tertiary care centre.

**Methods:**

This descriptive cross-sectional study was done among postmenopausal women in the outpatient department of a tertiary care centre from 10 March 2021 to 10 March 2022 after taking ethical approval from the Institutional Review Committee (Reference number: MEMG/IRC/420/ GA). Convenience sampling was done. Demographic data were collected using predesigned proforma and menopausal symptoms were evaluated using Rajanobritta Lakshan Mapak, the Nepali version of the menopausal rating scale. Point estimate and 95% Confidence Interval were calculated.

**Results:**

Among 424 postmenopausal women, menopausal symptom was found in 411 (96.48%) (94.73-98.23, 95% Confidence Interval). The mean age at menopause was 49±4.70 years. The most common symptom these women presented with was somatic symptoms seen in 382 (92.94%) women.

**Conclusions:**

The prevalence of menopausal symptoms is found to be higher than the other studies done in similar settings.

## INTRODUCTION

Menopause is defined as the complete cessation of menstruation for a consecutive 12 months.^1^ Natural menopause occurs due to the loss of follicular activity of ovaries from the late 40s to early 50s. The age of menopause ranges from 45 to 55 years worldwide with average onset at 51 years.^2,3^

During menopause, various symptoms like vasomotor symptoms, psychosexual and somatic symptoms arise due to withdrawal of hormones. These are aggravated by the ageing process which leads to a decrease in quality of life of the women after menopause.^4,5^ Only limited studies have been done to establish the prevalence of menopausal symptoms in our setting.

This study aimed to find the prevalence of menopausal symptoms among postmenopausal women visiting the outpatient department of a tertiary care centre.

## METHODS

A descriptive cross-sectional study was conducted among postmenopausal women in the outpatient department (OPD) of Gynaecology and Obstetrics, Manipal Teaching Hospital, Pokhara, Nepal from 10 March 2021 to 10 March 2022. Ethical approval was taken from Institutional Review Committee (IRC), Manipal College of Medical Sciences (Reference number: MEMG/IRC/420/GA). Postmenopausal women attending OPD for any reason were enrolled in the study. Women who did not consent to the study were excluded. Convenience sampling technique was used. The sample size was calculated using the following formula:


$$\begin{array}{ll} \text{n} & ={\text{Z}}^{2}\times \frac{\text{p}\times \text{q}}{{\text{e}}^{\text{2}}}\\ & =1.96^{2}\times \frac{0.50\times 0.50}{0.05^{2}}\\ & =385 \end{array}$$

Where,

n = minimum required sample size
Z = 1.96 at 95% confidence interval (CI)
p = prevalence taken as 50% for maximum sample size calculation
e = margin of error, 5%

The calculated sample size was 385. However, we have taken 424 postmenopausal women in our study. Face-to-face interview was done for the collection of data and was recorded in predesigned proforma and confidentiality of the information was maintained. The proforma included demographic profile, current age, age at menarche, age at menopause, marital status, state of parity, any medical disorders like diabetes, hypertension, neurological disorder, psychiatric disorder, thyroid disorder, and information pertaining to finding out whether it was natural or surgical menopause.

Symptoms of menopause were enquired based on the 11 symptoms as quoted in Rajanobritta Lakshan Maapak-Nepali version of the menopausal rating scale (MRS). This Nepali version of MRS was validated.^6^ MRS is a health-related quality of life scale developed in Germany (by The Berlin Centre for Epidemiology and Health Research) in the early 1990s.^7^ It is well accepted internationally which is composed of 11 items and was divided into three subscales like somatic, psychological, and urogenital symptoms.

Data were entered and analysed using Stata version 17. Point estimate and 95% CI were calculated.

## RESULTS

**Figure 1:**
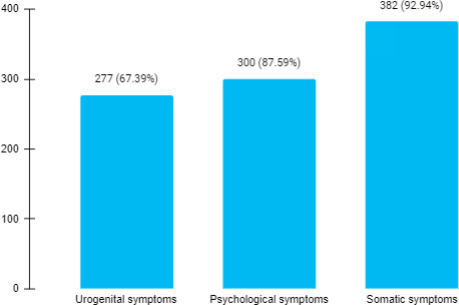
Frequencies of menopausal symptoms among postmenopausal women (n= 411).

Out of 424 postmenopausal women enrolled in the study, at least one menopausal symptom was found in 411 (96.48%) (94.73-98.23, 95% CI). On studying the menopausal symptom, it was found that the most prevailing menopausal symptom was somatic symptom which was present in 382 (92.94%) women (Figure 1). The mean age of women in the study group was 59.72±9.60 years. The mean age at menarche was 15.30±1.70 years and the mean age at menopause was 49±4.70 years.

Around 264 (64.23%) women were Brahmin and Chhetri and the majority 403 (98.05%) were married. Only 63 (15.32%) had a history of smoking. Among different medical morbidities studied, hypertension 136 (33.09%) was common. Only 41 (10%) women had surgical menopause (Table 1).

**Table 1:** Clinical profile of menopausal women with symptoms (n= 411).

Variables	n (%)
**Ethnicity**	
Brahmin and Chhetri	264 (64.23)
Janajati	103 (25.06)
Dalit	37 (9.00)
Madheshi	5 (1.21)
Others	2 (0.48)
**Marital status**	
Unmarried	8 (1.94)
Married	403 (98.05)
Smoking	63 (15.32)
**Parity**	
0	20 (4.86)
1 or 2	88 (21.41)
≥3	303 (73.72)
**Comorbidities**	
Diabetes	33 (8.02)
Hypertension	136 (33.09)
Neurological disorder	24 (5.83)
Thyroid disorder	49 (11.92)
Psychiatric disorder	12 (2.91)
Surgical menopause	41 (9.97)

## DISCUSSION

According to the literature, the experience of menopausal symptoms in menopausal women is said to be influenced by various factors which include biological, reproductive, social, psychological, and cultural factors. This might have led to the difference in the prevalence of menopausal symptoms across the globe.^8^

In our study, the prevalence of menopausal symptoms among menopausal women was 96.93%. In a study done in Sri Lanka, the prevalence of menopausal symptoms was like our study (96.4%).^9^ In different studies done in India, the prevalence of menopausal symptoms ranged between 78-89.3%,^10,11^ which is lower than the prevalence in our study. In a study done in Southern China, the prevalence of menopausal symptoms was low.^12^

It has been found that there has been a great variation in the age of menopause around the world. The mean age at menopause in our study was 49±4.7 years. In a study done in a tertiary care centre in Kathmandu, the mean age at menopause was also 49.9 years,^13^ similar to our findings. Studies done in different urban areas of Nepal have also shown similar ages at menopause.^5,14,15^ However, in studies done in rural districts, the mean age at menopause was higher (51.2-55.7 years).^16,17^

The studies done in India have shown that the mean age at menopause ranges between 45 to 52 years.^10,11,18,19^ In a systematic review regarding differences in age at menopause across the world, it has been found that the median age at menopause across Europe ranges from 50.1 to 52.8 years, in North America from 50.5 to 51.4 years, in Latin America from 43.8 to 53 years and in Asia from 42.1 to 49.5 years.^20^ So, these differences in mean age at menopause between countries provide us with an area to explore for factors responsible for causing these differences.

Postmenopausal women experience different menopausal symptoms related to somatic, psychological, and urogenital domains and it has been seen that every woman has her unique experience. Various tools have been developed to assess this experience of menopausal symptoms like the MRS, Menopause Specific Quality of Life Questionnaire (MENQOL), World Health Organization Quality of Life (WHO QOL-BREF), Greene Climacteric Scale, Women's Health Questionnaire (WHQ) and many others.^21^ In our study, the MRS tool has been used to assess the different menopausal symptoms. Using this scale in our study, it has been found that symptoms related to the somatic domain are the most prevalent ones followed by psychological and urogenital domains. A similar finding has been observed in other studies done in Nepal,^5,16,17^ as well as in a study done in India,^10-11-18^ Saudi Arabia,^22^ and Ethiopia.^23^ One study done in Nepal found urogenital symptoms to be more common followed by somatic symptoms.^13^ Psychological symptoms were more common in other studies.^11-14^

This was a cross-sectional study from a single centre so the results might not be generalizable in the large population. Future studies in the community setting is recommended.

## CONCLUSIONS

The prevalence of menopausal symptom among postmenopausal women was found to be higher than the similar studies done in similar settings. Majority of postmenopausal women experience menopausal symptoms and somatic symptoms were the common symptoms experienced by them. These symptoms can have impact in quality of life of postmenopausal women. All health-care providers and policymakers must be aware of and address this issue while dealing with postmenopausal women's problems and illnesses, as well as educate them about these symptoms and give the necessary support and treatment.
